# Vitamin D status and supplementation as potential modifiers of allergen immunotherapy outcomes in respiratory allergy: A structured narrative review

**DOI:** 10.1007/s12026-026-09854-3

**Published:** 2026-10-02

**Authors:** Selda Ali, Denisa-Mihaela Iordan, Andreea-Iulia Vlădulescu-Trandafir, Roxana Silvia Bumbăcea

**Affiliations:** 1 “Carol Davila” University of Medicine and Pharmacy, Bucharest, 050474 Romania; 2 Allergology Department, “Dr. Carol Davila” Nephrology Clinical Hospital, Bucharest, 010731 Romania; 3 Neuromuscular Rehabilitation Clinic Division, Teaching Emergency Hospital “Bagdasar- Arseni”, Bucharest, 041915 Romania

**Keywords:** Vitamin D, Allergen immunotherapy, Allergic rhinitis, Asthma

## Abstract

Allergen immunotherapy (AIT) represents a cornerstone in managing IgE-mediated allergic diseases, such as allergic rhinitis and asthma. By administering controlled doses of allergens over time, AIT aims to readjust immune tolerance, thereby modifying allergic responses. However, clinical responses to AIT remain heterogeneous, with a subset of patients achieving only partial benefit. This heterogeneity underlines the need for novel adjunctive approaches to potentiate the immunomodulatory effects of AIT. In this context, vitamin D has gained considerable interest, given its well-established role in immune homeostasis and regulation, as well as its emerging relevance in modulating allergic disease. This structured narrative review aims to assess the potential role of vitamin D supplementation during AIT on clinical outcomes as well as the significance of endogenous vitamin D levels on AIT clinical efficacy. A total of 18 studies were reviewed, 13 evaluating the impact of vitamin D supplementation during AIT and 5 assessing the influence of endogenous vitamin D levels on AIT outcomes. Most supplementation studies reported clinical outcomes favoring the addition of vitamin D to AIT, but three found no additional benefit, and two reported a benefit only at an earlier time point, respectively in vitamin D-deficient patients. Of the five studies examining endogenous status, three reported that higher 25-hydroxyvitamin D (25(OH)D) levels were associated with better AIT outcomes. Other findings that were reported as secondary outcomes included favorable immunologic changes, such as IL-10 production, decreased allergen-specific IgE levels, and enhanced regulatory Treg-cell activity, without formal assessment of their contribution to clinical benefit. Nevertheless, findings remain inconsistent across studies due to heterogeneity in study design, supplementation regimens, and outcome measures. While the available evidence suggests that adequate vitamin D status or supplementation may be associated with better clinical responses to AIT, the current findings remain limited by heterogeneity in study design, supplementation regimens, outcome measures, and generally small sample sizes. Therefore, the evidence should be considered suggestive rather than conclusive. Although correction of vitamin D deficiency is appropriate on general clinical grounds, its specific use as an adjunct to improve AIT outcomes cannot yet be routinely recommended. Further adequately powered, standardized, placebo-controlled studies are needed to clarify its role and inform clinical recommendations.

**Supplementary Information:**

The online version contains supplementary material available at https://doi.org/10.1007/s12026-026-09854-3.

## Introduction

### Allergen immunotherapy (AIT) in allergic rhinoconjunctivitis and asthma

Allergic rhinoconjunctivitis (AR) is an allergic condition with one of the highest prevalences among allergic disorders. It is characterized by predominant eosinophilic inflammation of the nasal mucosa and conjunctiva and can significantly impact patients’ quality of life. Asthma is a heterogeneous disease characterized by chronic airway inflammation and reversible airflow obstruction, allergic asthma being one of the most commonly encountered phenotypes, often accompanied by other allergic diseases such as allergic rhinitis, atopic dermatitis, or food allergy. Allergic inflammation is a Th2-driven inflammation that features an IgE-mediated mast cell activation and eosinophil and basophil recruitment and activation, leading to the secretion of specific inflammatory interleukins, such as IL-4, IL-5, IL-9, and IL-13 [1].

The standard pharmacotherapy used for treating AR and asthma, such as intranasal or inhaled corticosteroids (ICS), oral or topical antihistamines, or leukotriene receptor antagonists, aims to alleviate symptoms and decrease inflammation. Nevertheless, it is not always successful in reaching symptom control in some patients. In such circumstances, allergen immunotherapy (AIT) may be considered [2].

AIT is a disease-modifying treatment used to address IgE-mediated disorders such as allergic rhinitis and asthma, focusing on decreasing sensitization to a specific allergen, which subsequently leads to sustained reduction of allergic symptoms and, at the same time, reduces the economic burden of allergic diseases [3, 4]. The primary routes of administration for AIT are subcutaneous (SCIT) and sublingual (SLIT), both of which are effective in inducing immune tolerance and offering a long-term clinical benefit.

The mechanism of action of AIT involves the production of IgG4 antibodies that inhibit IgE-mediated reactions on effector cells such as mast cells, basophils, and B cells. It also implies a switch from a Th2-type immune response to a Th1-type immune response through the action of T regulatory (Treg) cells which suppress Th2 immunity and produce IL-10, thereby sustaining the inhibition of allergen-specific effector cells [3, 5].

### Assessment of AIT efficacy and the role of adjunctive therapies

#### Clinical parameters

AIT has been proven to effectively induce tolerance to specific allergens and provide long-term symptom alleviation even years after treatment completion [6]. Nonetheless, patients may exhibit varying responses to AIT, with differing levels of treatment efficacy, and usually, the efficacy of AIT is not predictable [7].

When referring to the assessment of AIT efficacy, several clinical parameters and in vitro biomarkers are used; however, some of these lack standardization and validation. The European Academy of Allergy and Clinical Immunology (EAACI) position papers on the standardization of clinical outcomes used in AIT for allergic asthma and allergic rhinoconjunctivitis, respectively [2, 8], present the clinical parameters used for assessing and quantifying the clinical improvement offered by AIT, along with their advantages and disadvantages.

Regarding the primary outcome measure for assessing the efficacy of AIT in treating allergic rhinitis, the EAACI task force (TF) has recommended using the Combined Symptom and Medication Score (CSMS) as it is a standardized method that equally evaluates symptoms as well as the necessity for antiallergic medication [8].

As clinically applicable quantification methods for assessing clinical outcomes in asthma, symptom scores and reduction in medication usage, such as ICS and reliever medication, are currently recommended for establishing the clinical outcomes of AIT. It has also been considered that a reliable objective primary outcome is represented by the exacerbation rate; however, due to the heterogeneity in the definitions used for exacerbations, evidence is still limited. Patient-reported outcomes measures (PROMs) such as the Control of Allergic Rhinitis and Asthma Test **(**CARAT), Asthma Quality of Life Questionnaire (ACLQ), or Asthma Control Questionaire-5 (ACQ5) are easy to perform and to assess disease control from the patients’ perspective; however, they are not regarded as primary endpoint parameters, and the relation between AIT and asthma control has yet to be validated. On the same note, symptoms and medication scores are useful for monitoring symptom control in patients undergoing AIT in daily practice; nevertheless, standardization remains necessary [8].

#### Immunological parameters

A biomarker is defined as a “characteristic that is objectively measured and evaluated as an indicator of normal biologic processes, pathogenic processes, or pharmacologic responses to a therapeutic intervention” (Biomarkers Definitions Working Group 2001) [9]. Biomarkers provide a means of quantifying clinical and immunological responses in patients undergoing AIT, aiding in the selection of appropriate patients for initiating AIT and in differentiating between future responders and non-responders. Although the predictability of AIT efficacy is not entirely feasible, several biomarkers have been used to monitor it. Among the most extensively studied biomarkers for assessing AIT efficacy, we find IgE (including total IgE (tIgE), specific IgE (sIgE) and the sIgE/tIgE ratio), IgG subtypes (specific IgG1, specific IgG4, and the sIgE/IgG4 ratio), IgE serum inhibitory activity (IgE-Facilitated Allergen Binding (IgE-FAB and IgE-Blocking Factor (IgE-BF)), basophil activation, cytokines and chemokines, cellular markers (T regulatory cells (Treg), B regulatory cells (Breg), and dendritic cells) and in vivo biomarkers such as nasal and chamber provocation tests [10].

Currently, there are no validated biomarkers that can predict the outcomes of AIT [5]. However, in the EAACI -TF position paper on the “Biomarkers for monitoring clinical efficacy of allergen immunotherapy for allergic rhinoconjunctivitis and allergic asthma”, a couple of them have been recommended as candidate biomarkers for clinical outcomes (sIgE/tIgE ratio and IgE-FAB), while sIgG4 was suggested as a biomarker for compliance [10]. More recent studies have brought insights regarding biomarkers and their utility in assessing AIT efficacy. A clinical commentary review conducted by Shamji et al. [11], summarized the biomarkers of AIT response, outlining that the biomarkers of AIT efficacy include sIgE/tIgE ratio, sIgG4, IgE-FAB, Treg cells, Breg cells, dendritic regulatory cells (DCreg), ILC10 and basophil activation. Nevertheless, they should not replace clinical outcome parameters.

### Vitamin D and the immune system

#### Vitamin D physiology, supplementation and role in immune function

Vitamin D is a liposoluble vitamin that can be found in two forms: vitamin D3 (also known as cholecalciferol) and vitamin D2 (also known as ergocalciferol). Both forms of vitamin D can be supplemented through diet, however, vitamin D3 is also produced within the skin from 7-dihydrocholesterol upon exposure to UVB radiation [12]. Vitamin D first undergoes hydroxylation in the liver under the action of 25-hydroxylase (CYP2R1) enzyme, forming the inactive form of vitamin D, namely 25-hydroxyvitamin D (25(OH)D), also known as calcidiol. 25(OH)D is currently regarded as the biomarker of assessing vitamin D status. 25(OH)D is further metabolized by 1a-hydroxylase enzyme into 1,25-dihydroxyvitamin D (1,25(OH)_2_D), also known as calcitriol, which represents the biologically active form of vitamin D [13]. 1,25(OH)_2_D exerts its actions through the vitamin D receptor (VDR), the general hormonal functions being related to bone health and mineral metabolism by the enhancement of calcium and phosphate absorption [14, 15].

While ideal vitamin D levels remain unclear and are still being debated in the literature, the optimal 25(OH)D levels indicated by the guidelines of the Endocrine Society fall within 20–30 ng/mL (50–75 nmol/L); other studies, on the other hand, have concluded that vitamin D sufficiency is within the range of 30–100 ng/ml. Vitamin D insufficiency is defined by levels between 20 and 29.9 ng/mL (52–72 nmol/L), and vitamin D deficiency by levels under 20 ng/mL (50 nmol/L) [16, 17].

In what concerns vitamin D supplementation, a couple of formulations currently exist, the most common being cholecalciferol (vitamin D3) and ergocalciferol (vitamin D2), but also others such as eldecalcitol, and calcifediol. Comparisons between the two more commonly used formulations, (cholecalciferol and ergocalciferol) have shown that cholecalciferol is more effective in raising 25(OH)D levels than ergocalciferol [12, 18]. Currently, there is no consensus describing an optimal regimen regarding vitamin D supplementation. Generally, the daily recommended vitamin D intake is between 400 and 800 IU, with 4000 IU being regarded as the safe daily intake limit, although the possibility of administering up to 10,000 IU has been assessed as safe in certain contexts. Yet, the ideal dose for each person may vary according to different factors such as therapeutic goal, age, weight etc. [12, 19].

Vitamin D also plays an important part in immune modulation because immune cells such as B and T cells, mast cells, macrophages, monocytes, and dendritic cells express VDR, having effects on both innate as well as adaptive immunity [13, 20]. The mechanisms by which vitamin D exerts its immunomodulatory effects show several unresolved issues, particularly regarding its influence on the Th1/Th2 balance and Th17/Treg balance, while the predominant regulatory pathway involved in the relationship between vitamin D and allergic diseases still requires further research [13, 15].

In T cell subsets, vitamin D usually acts by inhibiting Th1 and Th17 activity and promoting Th2 and Treg cell development. Within the macrophages, vitamin D reduces inflammatory responses by downregulating pro-inflammatory molecules such as IL-12, TNF-α, and IL-1β, and the co-stimulatory markers CD80 and CD86 in M1 macrophages, thus decreasing T-cell activation. At the same time, it increases IL-10 and TGF-β production in M2 macrophages, driving them toward a more anti-inflammatory phenotype. In dendritic cells, vitamin D reduces the expression of MHC class II and co-stimulatory molecules, thereby impairing their antigen presentation and the capacity for T cell activation. It also lowers levels of pro-inflammatory cytokines, such as TNF-α, IFN-γ, IL-2, and IL-23, favoring Th1 and Th17 differentiation while upregulating IL-10 to support anti-inflammatory responses [13].

However, in B cells, vitamin D has shown inhibitory effects on proliferation, also suppressing plasma cell differentiation and IgE production. It has also been associated with maintaining mast cell stability and limiting IgE-mediated mast cell release of inflammatory and vasodilatory mediators, as well as enhancing Treg formation by promoting FOXP3 transcription. Vitamin D also prevents the production of cytokines like IL-5 and IL-13 by inhibiting innate lymphoid cells 2 (ILC2). Regarding eosinophils, vitamin D facilitates their migration from inflammation sites to non-inflammatory tissues while also reducing the production of eosinophil mediators such as major basic protein (MBP), eosinophil peroxidase (EPX), eosinophil cationic protein (ECP), and eosinophil-derived neurotoxin (EDN) [13, 21, 22].

#### Role of vitamin D in allergic rhinoconjunctivitis and asthma

There is currently conflicting evidence regarding the relationship between vitamin D and allergic rhinitis and asthma. Vitamin D has been shown to favorize the shift towards the Th2 mechanism, while suppressing the differentiation and activation of Th1 and Th17 cells, also modulating IgE production and B cell activity, therefore playing a role in the promotion of immune balance [14]. Regarding AR, some studies suggest a reduction in AR symptoms and prevalence as well as a reduction in asthma severity, while others have not correlated a significant relationship, or even associated vitamin D supplementation with a higher AR prevalence [21]. A recent systematic review and meta-analysis conducted by Li et al. [23] concluded that a reduction in AR symptom severity in children could be reached by vitamin D supplementation.

Regarding asthma, it has been shown that vitamin D exhibits protective effects by influencing smooth muscle function and airway remodeling by inhibiting the growth of airway smooth muscle through various mechanisms. These findings highlight the potential of vitamin D in regard to the management of asthma, however, conflicting findings such as in vitro studies that suggest a Th2 shift promoted by vitamin D have led to the contradiction in what concerns vitamin D’s protective role. From a clinical point of view, it has been determined that vitamin D supplementation may help in the reduction of asthma exacerbations in children with 25(OH)D levels < 10 ng/mL, however, this cannot be generalized to all children. Most research supports the association between higher vitamin D levels and reduced asthma severity; however, evidence remains inconsistent. Therefore, further research is necessary to better understand the role of vitamin D in asthma management and to determine safe, effective dosing regimens for those most likely to benefit [13].

## The role of vitamin D in AIT for allergic rhinoconjunctivitis and asthma

### Materials and methods

This is a structured narrative review that aimed to explore the influence of vitamin D (whether supplemented or endogenously sufficient) on the clinical outcomes of AIT in patients with allergic rhinitis and/or asthma. The review was conducted using a focused yet flexible approach to evidence synthesis, which was considered appropriate for the heterogeneity of the current literature.

The literature search was conducted across five major academic databases: PubMed, Web of Science (WOS), ScienceDirect, Cochrane Library and Scopus, covering publications from 1st of January 2005 to 10th of August 2026, with the date of the last search being 12th of August. The start date was selected to capture the growing body of evidence that emerged in the mid-2000s, when the immunomodulatory role of vitamin D in allergic diseases and its potential as an adjunct to AIT began to be clinically investigated. Older literature was excluded to ensure relevance to modern immunotherapy protocols and vitamin D assessment standards.

To improve sensitivity and reproducibility, the search combined keyword syntaxes related to vitamin D with terms related to AIT. The vitamin D concept included “vitamin D”, “vitamin D2”, “vitamin D3”, “cholecalciferol”, “ergocalciferol”, “calcifediol”, “calcidiol”, “calcitriol”, “25-hydroxyvitamin D”, “25(OH)D” and “25OHD”. The AIT concept included “allergen immunotherapy”, “allergen-specific immunotherapy”, “allergy immunotherapy”, “specific immunotherapy”, “subcutaneous immunotherapy”, “sublingual immunotherapy”, “SCIT”, “SLIT”, and “desensitization”.

The strategy was adapted to the indexing system, available fields and syntax of each database. In PubMed, MeSH terms were combined with free-text terms searched in titles and abstracts. Scopus was searched using the TITLE-ABS-KEY field. In Web of Science Core Collection, the Topic field was used, covering titles, abstracts, Author Keywords and Keywords Plus. Because ScienceDirect allows a maximum of eight Boolean connectors within each search field, the strategy was divided into four complementary searches. In the Cochrane Library, MeSH descriptors were combined with terms searched in titles, abstracts and keywords. No document-type or open-access filters were applied. Publication type was evaluated during screening because database classifications are not always consistent and relevant original clinical data may be mistakenly indexed as a different type of publication. We provided the complete search strings, fields searched, limits, search dates and numbers of records retrieved from each database in Supplementary Table S1.

The search was restricted to English-language reports because resources for the reliable translation and assessment of non-English full texts were not available. When interpreting the findings, we considered the possibility of language bias arising from this restriction.

Studies were considered eligible if they included patients with allergic rhinoconjunctivitis and/or asthma undergoing AIT and examined either: (1) vitamin D supplementation administered as an adjunct to AIT, or (2) the relationship between endogenous vitamin D status and clinical response to AIT. Eligible studies were required to report at least one clinical outcome related to AIT efficacy, such as symptom control, medication use, disease control, or response to allergen challenge. Immunological outcomes, including IL-10, allergen-specific IgE and regulatory T-cell activity, were considered as secondary mechanistic outcomes when reported alongside the clinical findings. The exclusion criteria consisted of studies that: (1) included patients who did not undergo AIT for allergic rhinitis/rhinoconjunctivitis and/or asthma (2) analyzed data from animal models, (3) did not analyze vitamin D-dependent changes in AIT clinical outcomes, (4) reported biomarker findings without corresponding clinical outcomes. Reviews, meta-analyses, practice guidelines, consensus papers, and editorials were excluded from the clinical synthesis and were used only to provide background information or to identify additional primary studies. Case reports were also excluded.

Full-text original clinical studies conducted in human participants formed the primary evidence base of the review. Conference abstracts reporting original clinical data were also retained as supplementary evidence when they described relevant patient cohorts or outcomes for which no full-text publication could be identified. Given their limited methodological and outcome reporting, conference abstracts were considered separately from full-text studies and were not given equivalent weight in the interpretation of the evidence. When an abstract and a full-text publication were found to report the same study population, the full-text report was prioritized to avoid double counting.

**Fig. 1:**
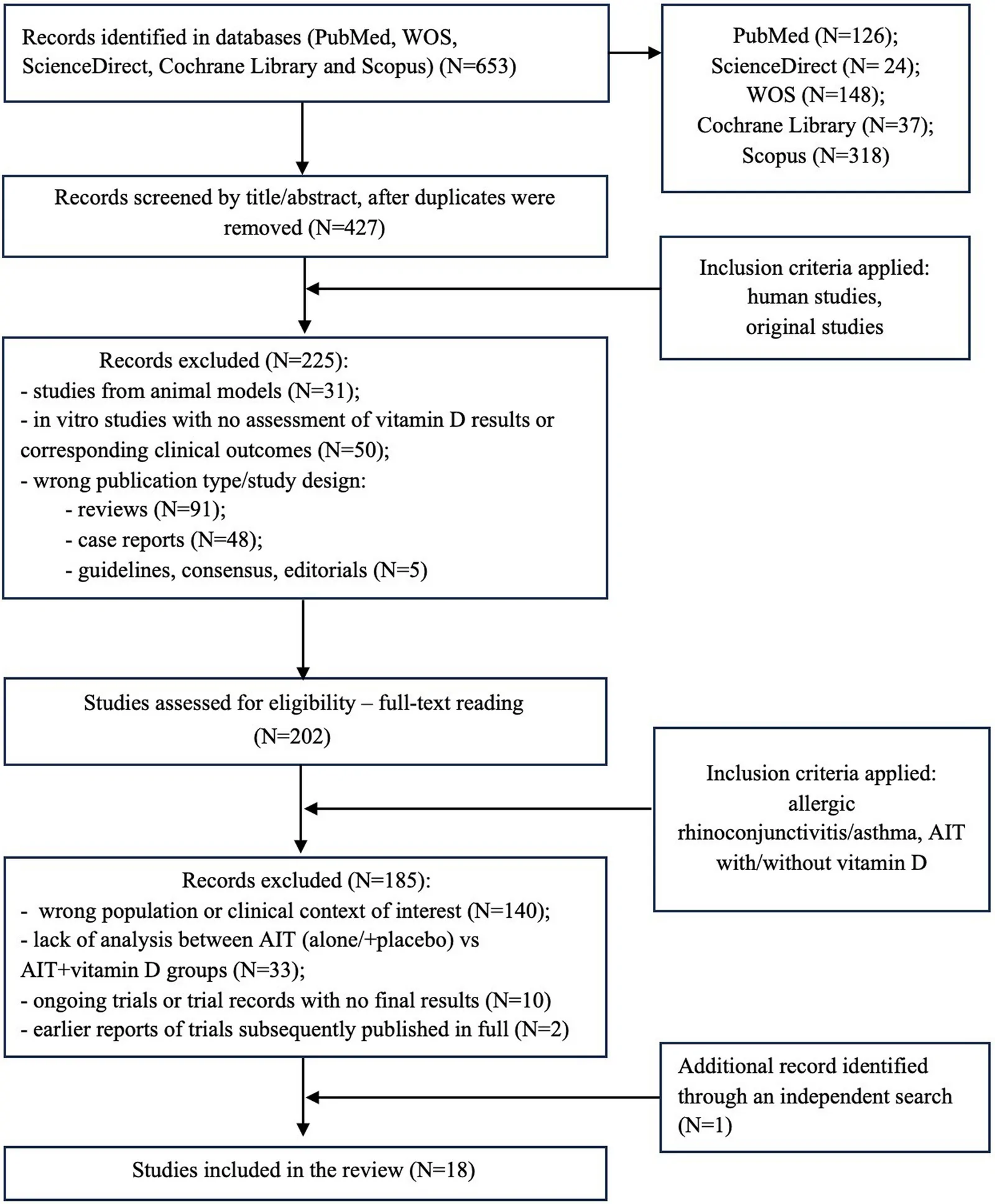
Flowchart of the literature search process

The updated search retrieved 653 records (Fig. 1): 126 from PubMed, 24 from ScienceDirect, 148 from WOS, 37 from Cochrane Library and 318 from Scopus. Two authors independently screened the titles and abstracts using the Rayyan platform [24], using the eligibility criteria described above and any divergence was resolved by consulting with a third author. After eliminating 226 duplicates, 427 records remained. Subsequently, 144 were excluded because of an ineligible publication type or study design, including reviews, case reports, guidelines, consensus papers, and editorials. A further 81 records were excluded because they reported animal or in vitro research with no assessment of vitamin D results or corresponding clinical outcomes. The remaining 202 records underwent a more detailed assessment based on the full text. Of these, 140 did not address the population or clinical context of interest, 33 did not evaluate vitamin D-related changes in clinical AIT outcomes or reported biomarker findings without corresponding clinical outcomes, and 10 were ongoing trials or trial records for which no final results were available. Additionally, 2 records were identified as being the same trials at different stages of publication and we retained only the corresponding full publication. Finally, 17 publications were retained for the narrative synthesis. One additional eligible record, a doctoral thesis chapter, was identified through an independent online search and was included to provide a more comprehensive overview of the available evidence, resulting in a total of 18 publications included in the review. The literature search and selection process for this structured narrative review are summarized in Fig. 1.

## Results

Data were extracted from the search characteristics and key features were included in Tables 1 and 2. Table 1 includes studies that analyzed the role of vitamin D supplementation during AIT on the efficacy of AIT. It encompasses general study characteristics such as the type of study conducted, number of patients included, age group, diagnosis and allergen sensitization, type of AIT, vitamin D regimen (form, dose, route of administration and duration), the timing of outcome assessment, baseline and achieved 25(OH)D concentrations, the main clinical findings for the comparison of AIT plus vitamin D versus AIT alone, and the overall clinical benefit reported. Table 2 includes studies that analyzed the difference in AIT outcomes according to the endogenous vitamin D level. Similarly, it follows the same format, with patients grouped according to their vitamin D levels rather than a supplementation regimen. Both tables focus on clinical outcomes, while the secondary immunological findings are discussed separately in Section 3.1.3.

**Table 1:** Reported results of vitamin D supplementation during AIT for allergic rhinitis and/or asthma

Author, year	Study type; number of patients	Population (age group; diagnosis; allergen)	AIT type	VD intake (form; dose; route; duration)	Time of results analysis	25(OH)D baseline → achieved levels in AIT + VD group	Main clinical findings (AIT + VD vs. AIT alone)	Overall clinical benefit of VD supplementation during AIT
Studies with complete methods and outcome data								
Baris S et al., 2014ᵃ [25]	RCT; 32 (of 50)	Children; asthma +/- AR; HDM	SCIT	VD3; 650 IU/day; oral; 12 months	6 months and 1 year	19 ± 9 → 31.2 ± 10.1 ng/mL	TASS lower with VD at 6 months (*P* = 0.01); no difference in symptom/medication scores at 12 months; ICS discontinued in 6/17 vs. 3/15, P value NR; annual asthma attacks 1.6 ± 1.3 vs. 2.1 ± 1.3, P value for between-IT-group comparison NR	Benefit reported at 6 months only
Van Hemelen D, 2016* [26]	RCT, double-blind, placebo-controlled; 54	Adults; AR +/- asthma; Grass pollen	SCIT (allergoid)	Calcitriol; 0.04 µg/kg; subcutaneous; 49 weeks	9 weeks and 49 weeks	34 → 71 (week 9); 36 (week 49) nmol/L	No significant additional clinical benefit of VD over AIT alone across nasal provocation, symptoms and other clinical measures	No benefit reported
Jerzynska J et al., 2016 [27]	RCT, double-blind, placebo-controlled; 50	Children; AR +/- asthma; Grass pollen	SLIT	VD3; 1000 IU/day; oral; 5 months	5 months	48.8 ± 5.3 → 94.8 ± 6.8 ng/mL	CSMS: 43.7 vs. 57.8 (*P* = 0.001); nasal symptoms *P* = 0.04; asthma symptoms *P* = 0.001; 28% CSMS reduction (> 20% clinical relevance threshold). Medication score not different (*P* = 0.21)	Benefit reported
Yu ZJ et al., 2017 [28]	RCT, placebo-controlled; 40 (of 80)	Adults; AR +/- asthma; HDM	SCIT	VD3; 2000 IU/day; oral; 6 months	6 months	20–30 ng/mL (range) → NR	Lower symptom and medication scores with AIT+VD3 vs. AIT alone (*P* < 0.05); numerical between-group estimates NR	Benefit reported
El-Maghraby HM et al., 2019 [29]	Non-RCT; 48	Adults; AR; Date palm pollen	SCIT	VD3; 50,000 IU/week × 3 months, then 2000 IU/day × 3 months; oral	6 months	“Moderate deficiency” (value NR) → NR	Greater reduction in TNSS at 6 months with AIT + VD: 4.04 ± 1.33 vs. 7.58 ± 2.15 (*P* < 0.001), from comparable baselines (11.0 ± 2.1 vs. 11.2 ± 2.1)	Benefit reported
Heine G et al., 2020 [30]	RCT, double-blind, placebo-controlled pilot; 36	Adults; AR +/- asthma; Grass pollen	SCIT	VD3; 5333 IU/day; oral, November–April × 3 consecutive years	1 year, 2 years and 3 years	40.4 → 118.9 (year 1); 146.5 (year 2); 129.2 (year 3) nmol/L	CPT comparable between groups (*P* = 0.38); SMS similar in year 1; years 2–3 not analyzed (< 50% diary recovery)	No benefit reported
El-Korashi L et al., 2021 [31]	RCT, single-blind, controlled; 46 (of 69)	Children; asthma +/- AR; Different inhalant allergens	SCIT	VD3; 600 IU/day; oral; 6 months	6 months	10.3 (10–25) → 30 (19–35) ng/mL	ICS discontinuation: 56.5% vs. 21.7% with AIT alone (*P* = 0.016)	Benefit reported
Izmailovich MR et al., 2022 [32]	Open-label RCT; 51	Adults; AR; Different pollens	SLIT	VD3; individualized to baseline status; target 40 ng/mL (doses NR); route NR; ~8 months	VD levels − 2.5 months; TSSS − 8 months	16.32 (13.21–24.33) → 37.6 (32.4–41.06) ng/mL	TSSS reduction: 46% vs. 22% (final score 6 vs. 9; *P* = 0.041)	Benefit reported
Chiewchalermsri C et al., 2023ᵇ [33]	RCT, double-blind, placebo-controlled; 34	Adults; AR +/- asthma; HDM	SCIT	VD2; 60,000 IU/week; oral; 10 weeks	10 weeks and 20 weeks	20.36 ± 6.04 → 41.91 ± 8.01 (week 10); 24.86 ± 3.84 (week 20) ng/mL	No significant benefit in the whole cohort; in VD-deficient patients, SMS improved significantly with VD at weeks 10 (MD − 54.54%, *P* = 0.007) and 20 (MD − 42.69%, *P* = 0.04)	Benefit reported in VD-deficient patients only
Di Gioacchino M et al., 2023 [34]	Post-hoc analysis; 40	Children; AR +/- asthma; HDM	SLIT (allergoid)	VD3; 400–1000 IU/day; oral; prescribed by the treating pediatrician outside the study protocol; 12 months	1 year	20.1 ± 4.2 → 36.1 ± 2.8 ng/mL	ARIA score: 0.9 vs. 1.5; VAS: 1.7 vs. 3.2; antihistamine use: 0/12 vs. 5/28 (all *P* < 0.05)	Benefit reported
Conference abstracts								
De Cordova JF, 2016 [35]	RCT, placebo-controlled; 60	Children; AR; Allergen NR	SCIT	VD3; 1600 IU/day; oral; 3 months	3 months	NR → NR	CARATKids, TNSS and ARIA classification significantly better with AIT + VD; numerical values and P values NR	Benefit reported; no between-group P value
Sudiro M et al., 2018 [36]	RCT, double-blind, placebo-controlled; 36	Adults; AR; Allergen NR	SCIT	Calcitriol; 0.5 µg/day; oral; 15 weeks	8 weeks and 15 weeks	“deficiency” (value NR) → NR	NSS did not differ significantly betweengroups (*p* > 0.05)	No benefit reported
Kolesnykova O et al., 2024 [37]	Study type NR; 150	Age group NR; AR; Ragweed pollen	NR	NR	1 year	NR → NR	VAS improved in both groups (7.9→1.8 vs. 7.4→3.5; *P* < 0.05 within groups); medication use decreased significantly only with VD; between-group VAS comparison NR	Benefit reported; no between-group P value

“AR” - allergic rhinitis; “AIT” - allergen immunotherapy; “RCT” - randomized controlled trial; “HDM” - house dust mite; “SCIT” - subcutaneous immunotherapy; “SLIT” - sublingual immunotherapy; “VAS” - visual analogue scale; “ARIA” - allergic rhinitis and its impact on asthma; “TSSS” - total symptom severity score; “TNSS” - total nasal symptom score; “NSS” - nasal symptom score; “TASS” - total asthma symptom score; “SMS” - symptom medication score; “CSMS” - combined symptom medication score; “CPT” - conjunctival provocation test; “VD” - vitamin D; “VD2” - ergocalciferol; “VD3” - cholecalciferol; “25(OH)D” − 25-hydroxyvitamin D; “ICS” - inhaled corticosteroids; “CARATKids” - Control of Allergic Rhinitis and Asthma Test for Children Score; “NR” - not reported; “MD” - mean difference;

*Thesis chapter

ᵃ, ᵇPreviously reported at an earlier stage as conference abstracts [38] and [39]; each pair is counted as one study

**Table 2:** Reported results on the role of endogenous vitamin D levels on the outcome of AIT for allergic rhinitis and/or asthma

Study characteristics							VD-dependent differences in AIT outcomes
Author, year	Study type; number of patients	Population (age group; diagnosis; allergen)	AIT type	VD level groups (ng/mL)	Time of result analysis	Main clinical findings	Association between higher VD level and AIT outcome
Majak P et al., 2012 [40]	Post-hoc analysis from 2 prospective placebo-controlled RCT; 36	Children; Asthma; HDM	SCIT	> 30 vs. ≤ 30	3 months and 12 months	Higher 25(OH)D at 3 months associated with greater ICS dose reduction (*r* = − 0.64) and greater improvement in asthma symptom score (*r* = − 0.67) at 12 months; more patients with > 50% ICS dose reduction when 25(OH)D > 30 ng/mL at 3 months (*P* = 0.025)	Association reported
Joudi M et al., 2019 [41]	Cross-sectional study; 55	Adults; AR; Different pollens	SCIT	> 30; 20–30; 10–19; < 10	1 year	With < 10 vs. > 30 ng/mL: SNOT-22 reduction 18.3 vs. 39.0 and MiniRQLQ reduction 17.4 vs. 30.7 (*P* < 0.001)	Association reported
Jia X et al., 2022 [42]	Observational study; 62 (of 76)	Children; Asthma +/- AR; HDM	SCIT	> 20; 12–20; <12	1 year	All groups improved significantly (*P* ≤ 0.043); no difference between groups in symptom (*P* = 0.409) or medication scores (*P* = 0.235)	No association reported
Li L et al., 2023 [43]	Retrospective cohort; 101	Children; AR +/- asthma; HDM	SLIT	> 22.25 vs. ≤ 22.25	6 months	Higher response (CSMS improvement > 50%) in 94.3% in > 22.25 ng/mL group vs. 57.6% in ≤ 22.25 ng/mL group (*P* < 0.001); adjusted OR 22.21 (95% CI 4.04–122.30; *P* < 0.001)	Association reported
Petrarca C et al., 2023 [44]	Post-hoc analysis; 60 (of 165)	Children; AR +/- asthma; HDM	SLIT (allergoid)	≥ 27 vs. < 27	12 months	VAS 1.7 vs. 3.2 and oral antihistamine use 0% vs. 21% in ≥ 27 ng/mL group vs. < 27 ng/mL group (both *P* < 0.0001); no difference in ARIA, ACT or ICS-LABA	Association limited to VAS and antihistamine use

“AR” - allergic rhinitis; “HDM” - house dust mites; “AIT” - allergen immunotherapy; “SCIT” - subcutaneous immunotherapy; “SLIT” - sublingual immunotherapy; “VAS” - visual analogue scale; “CSMS” - combined symptom medication score; “RCT” - randomized controlled trial; “VD” - vitamin D; “SNOT-22” - Sino-nasal Outcome Test-22; “MiniRQLQ” - Mini Rhinoconjunctivitis Quality of Life Questionnaire; “ICS” - inhaled corticosteroids; “LABA” - long-acting beta-agonist; “ARIA” - allergic rhinitis and its impact on asthma; “ACT” - asthma control test; “OR” - odds ratio; “CI” - confidence interval; “25(OH)D” − 25-hydroxyvitamin

Our review includes 18 records (15 articles and 3 conference abstracts); 13 of them assess vitamin D-dependent differences in AIT outcomes in patients who received vitamin D during AIT versus patients undergoing AIT alone or AIT with placebo (Table 1), and 5 of them assess the association of endogenous levels of vitamin D with AIT outcomes (Table 2).

### The role of vitamin D supplementation on AIT outcomes

#### Study characteristics

The 13 reviewed studies included 10 randomized controlled trials [25–28, 30–33, 35, 36], as well as 1 non-randomized controlled trial [29] and 1 post-hoc analysis [34]. In one conference abstract, the study design was not specified [37]. Five of these included children [25, 27, 31, 34, 35] seven included adults [26, 28–30, 32, 33, 36] with allergic rhinitis/rhinoconjunctivitis and/or asthma, and in one of the studies, the age category was not specified [37]. Two pairs of records referred to the same trials at different stages of publication: the conference abstract by Safa et al. [38], and the subsequent full report by Baris S et al. [25], as well as the abstract by Chiewchalermsri C et al. [39] and its corresponding full publication [33]. To avoid double counting, each pair was treated as a single study and included only once in Table 1, based on the date from the full report.

Allergen-specific AIT was conducted for HDM in 4 of the studies [25, 28, 33, 34], grass pollen in 3 studies [26, 27, 30], date palm pollen in one study [29] and ragweed pollen in one study [37]. Two studies focused on AIT for various inhalant allergens [31], and respectively, for various pollens [32], while two did not mention the specific sensitizations treated [35, 36]. The sensitizations were assessed using skin prick tests and/or specific IgE measurement.

Most studies implied SCIT, (9 studies) [25, 26, 28–31, 33, 35, 36, 39], while three studies used SLIT [27, 32, 34]. In one study, the type of AIT was not specified [37]. Two of the studies employed allergoid AIT, one for grass pollen [26], and one for HDM [34].

Baseline vitamin D levels were measured in most studies, with results being heterogeneous. We considered baseline vitamin D levels in groups receiving both AIT and vitamin D. Five studies documented mean or median values < 20 ng/ml, indicating vitamin D deficiency [25, 26, 30–32]. Three studies reported levels within the range of 20–30 ng/ml, indicating vitamin D insufficiency [28, 33, 34]. However, in the study by Chiewchalermsri C et al. [33], 9 of the 15 patients were vitamin D-deficient. Two studies noted “vitamin D deficiency” and “moderate vitamin D deficiency” without providing specific values [29, 36]. The only study that reported a vitamin D level consistent with vitamin D sufficiency is the one conducted by Jerzynska J et al. [27], with baseline levels of 48.8 ± 5.3 ng/mL, which is significantly higher than the other studies. Two studies did not provide data about vitamin D status [35, 37]. Generally, there were no significant intergroup differences in baseline vitamin D levels. Except for the study reported by Jerzynska J et al. [27], participants generally had deficient or insufficient vitamin D levels at baseline, and in two studies the population was described as vitamin D-deficient without specific values being reported [29, 36]. The findings should therefore be interpreted mainly in the context of vitamin D-deficient or -insufficient populations and may not apply to patients who already have sufficient vitamin D levels when AIT is initiated.

The dose and duration of vitamin D supplementation during AIT varied widely across studies. Seven studies used daily doses ranging from 400 to 1000 IU/day [25, 27, 31, 34], 1000–2000 IU/day [28, 29, 35], to 5333 IU/day [30]. Di Gioacchino M et al [34] used pediatrician-prescribed daily doses of 400–1000 IU/day outside the study protocol. One study used a weekly regimen, the patients receiving 60 000 IU/week for 10 weeks [33]. Another study implemented an initial high-dose protocol of 50 000 IU/week for 3 months, decreasing the dose to 2000 IU/day for another 3 months [29]. One study implemented a weight-adjusted protocol, administering 0.04 µg/kg subcutaneously for 49 weeks, making it the only study to use injectable vitamin D [26]. Another study adopted a tailored approach to vitamin D administration based on each patient’s baseline vitamin D levels, with a target final level of 40 ng/ml; however, it did not mention the actual doses administered [32]. Likewise, one additional study did not specify the dose of vitamin D administered [37].

Supplementation duration also varied significantly, ranging from short-term regimens of 10 weeks [33] to longer ones, with most participants supplementing for 3–12 months [25–29, 31, 32, 34–38]. One study described a supplementation from November to April for 3 consecutive years [30]. Regarding the form of vitamin D administered, the majority of the studies supplemented with vitamin D3, except for the one conducted by Chiewchalermsri C et al. [33], which utilized vitamin D2, and two studies in which calcitriol was administered, orally at 0.5 mcg/day by Sudiro M et al. [36] and subcutaneously by Van Hemelen D [26].

Following vitamin D supplementation, most studies showed improved vitamin D status in groups receiving AIT and vitamin D, with the majority reaching levels > 30 ng/ml. In 6 of the studies, values between 30 and 50 ng/ml were reached [25, 30–34], while one of those reported a sustained improvement over the course of 3 years, obtaining values of 47.6 ng/mL, 58.7 ng/mL, and 51.8 ng/mL at years 1, 2, and 3, respectively [30]. In two studies, concentrations rose initially and declined thereafter. [26, 33]. The highest levels (94.8 ± 6.8 ng/ml) were observed in the study by Jerzynska J et al., despite the administered dose not being the highest among the studies [27]. Five of the studies did not report vitamin D levels. [28, 29, 35–37]. Overall, most studies showed a significant increase in vitamin D levels.

Vitamin D safety was assessed in 7 out of 13 supplementation studies [25–27, 30, 31, 33, 34]. One additional study noted only that no participant had 25(OH)D levels above 50 ng/ml [28], while the other five did not provide safety information. Calcium and phosphorus were monitored in four studies [25–27, 31], parathyroid hormone in two [25, 31], and calcium alone in one [33], with no abnormal values being reported. Normal safety parameters were also reported in the study using the highest vitamin D dose, 5333 IU/day over three years [30]. The only metabolic change observed was a significant decrease in serum phosphorus levels in the study conducted by Jerzynska J et al. [27], which also had the highest achieved mean 25(OH)D level (94.8 ng/mL); no sign of hypervitaminosis and no cases of hypercalcemia were reported. Adverse events were mostly related to AIT itself, including local injection-site reactions [25, 26, 31], sublingual itching [27], and mild asthma attacks [25, 31], and occurred in both study groups where this was reported. The only systemic reaction requiring epinephrine occurred in the AIT-only group [25], and no treatment discontinuations were attributed to vitamin D. However, the clinical significance of high 25(OH)D concentrations remains unclear. In addition, most studies provided limited safety data, and the longest reported exposure was three years [30], making it difficult to draw conclusions about long-term safety.

#### Clinical results of the studies

Each study analyzed the clinical benefit of vitamin D supplementation during AIT for allergic rhinitis and/or asthma by comparing groups that received AIT and vitamin D with groups that received either AIT alone or AIT and placebo, thereby assessing whether there are any vitamin D-dependent improvements in AIT efficacy. Apart from the baseline clinical evaluation, the groups’ second, and in some cases third and fourth evaluations were carried out at different time points in each study, therefore creating differences in study design. Most studies focused on a single post-treatment assessment, with time frames varying significantly between them. Among these studies, the earliest assessment was carried out at 3 months in the study by De Cordova JF et al. [30], and the latest took place after one year in the studies conducted by Di Gioacchino M et al. [34] and Kolesnykova O et al. [37]. The others assessed at intervals between 5 and 8 months [27–29, 31, 32]. Other studies evaluated at multiple time points, with some focusing on earlier assessments, such as at 10 and 20 weeks in the study by Chiewchalermsri C et al. [33], and at 8 and 15 weeks in the study by Sudiro M et al. [36]. Others focused on evaluating at later time points like 6 and 12 months in the study by S Baris et al. [25] and at 1 year, 2 years, and 3 years in the study by Heine G et al. [30]. Another study combined an early assessment with evaluations after longer periods, such as the study conducted by Van Hemelen D [26] (which assessed patients at 9 weeks and at 49 weeks).

Among the 13 supplementation studies, 6 reported a clinical benefit of adding vitamin D to AIT compared to those receiving only AIT alone or with placebo [33, 35, 37, 40, 41, 43]. Two studies showed a partial benefit of AIT + vitamin D: Baris et al. found lower total asthma symptom scores at 6 months, but no difference in symptom or medication score at 12 months [25]. Chiewchalermsri et al. found no benefit in the whole cohort but a significant symptom–medication scores’ improvement among vitamin D-deficient patients [33]. Meanwhile, three studies concluded that vitamin D offered no additional benefit to AIT [26, 30, 36]. The remaining two were conference abstracts reporting improvement in favor of the vitamin D group without between-group statistical comparisons [35, 37].

Improvements in AIT efficacy in the AIT + vitamin D groups compared to AIT alone or AIT + placebo groups were observed across various clinical parameters, as each study opted for different methods of measuring clinical efficacy, such as various symptom scores and the reduction in the use of symptomatic medication like antihistamines, intranasal or inhaled corticosteroids. Improvements in asthma-related outcomes, including increased inhaled corticosteroid discontinuation rates, were noted [25, 31], as well as improved asthma symptoms [27]. However, one study reported a favorable result only at 6 months after treatment, not at 12 months [25]. Combined symptom medication scores were used in two studies and showed significant improvement in favor of the AIT + vitamin D group compared to the AIT + placebo groups [27, 33]. In addition to this, one of the studies also reported a higher treatment response rate [33], and the other study reported improvements in asthma and nasal symptoms [27]. Significant differences in nasal symptoms were observed across 4 of the studies [27–29, 35], as evidenced by reduced nasal symptom scores in 3 [28, 29, 35], one only indicating the improvement of nasal symptoms [27]. Medication use also decreased, specifically oral antihistamines, nasal and inhaled corticosteroids [31 ,34, 37]. Other parameter changes such as visual analogue scale (VAS) score decrease [34, 37], ARIA classification improvement [34, 35] and improvement in the control of allergic rhinitis and asthma test for children score (CARATKids) [35] further supported the enhanced efficacy of AIT in patients supplemented with vitamin D. These findings collectively are consistent with a possible additional benefit of vitamin D supplementation during AIT for allergic rhinitis and/or asthma. However, the magnitude of these benefits varies depending on multiple factors, as the studies differ in design, endpoints and follow-up, and effect sizes are rarely accompanied by confidence intervals or adjustment for multiple comparisons.

Three studies concluded that there was no clinical benefit from supplementing with vitamin D during AIT. Therefore, Van Hemelen D [26] reported no significant differences between VAS scores and peak nasal inspiratory flow after nasal provocation testing and clinical index score, as well as the combined symptom medication scores during the grass pollen season. Heine G et al. [30] concluded that there were no significant differences between titrated conjunctival provocation tests (CPT) and symptom medication scores (SMS), while Sudiro M et al. [36] found no significant differences between NSS using the VAS scale.

Reported outcomes did not show a clear pattern in relation to vitamin D exposure. Neither higher achieved vitamin D levels nor longer supplementation appeared to translate into greater clinical benefit: clinical improvement was reported at concentrations from around 30 ng/mL to over 90 ng/mL and after supplementation periods as short as ten weeks, while the study with the highest levels and the longest duration reported none [30]. Both studies using calcitriol, given orally [36] and subcutaneously [26], reported no additional benefit, whereas the remaining studies that used oral vitamin D3 or D2 showed mixed results. Differences were also shown across clinically relevant subgroups. All three studies using SLIT reported a clinical benefit [27, 32, 34], whereas findings from the nine SCIT studies were less consistent. Four of the five pediatric studies showed a clinical benefit [27, 31, 34, 35], while results in adults were mixed. Among the three grass-pollen studies, benefit was reported only in the SLIT study [27]. Of the four house dust mite studies, two reported a benefit [28, 34], while two found an effect only in a subgroup or at a single time point [25, 33]. These comparisons should be interpreted cautiously, as most subgroups include only two to four studies and differ in vitamin D regimens, outcome measures, and follow-up periods. The subgroups also overlap, so these findings are best considered descriptive rather than conclusive.

#### Immunological changes during AIT - results from the studies

As secondary outcomes of the studies, some immunological parameters were also measured, and differences between the AIT + vitamin D groups versus AIT alone or with placebo were noted, with a focus on the improvements within the AIT + vitamin D group compared with the other groups. Among studies that measured differences in specific IgE levels between groups, 5 reported decreased specific IgE levels in favor of the AIT + vitamin D group [25, 28, 29, 34, 37]. In contrast, 2 studies reported no significant differences in specific IgE levels after treatment between groups [26, 32], while the remaining studies either did not measure specific IgE levels or did not report comparisons between the groups.

Seven studies also measured IL-10 levels, which were notably increased in the AIT + vitamin D group in 4 of the studies [28, 29, 31, 36]. Specifically, in the study conducted by Sudiro M et al. [36], a more rapid increase in IL-10 levels at week 8 was noticed in the group supplemented with vitamin D, compared to week 15 in the group that did not receive vitamin D. In regard to IgG4 level increase, most studies either did not observe significant differences between groups [25, 26] or did not measure it. In contrast, one study noted an increase in the IgG4/IgE ratio in favor of the AIT + vitamin D group after the first AIT course [30]. In addition to these parameters, some studies measured additional immunological markers. The other most commonly measured markers were related to Treg cells. Respectively, vitamin D supplementation was associated with increased CD4 + CD25+FOXP3 + Treg cells with increased FOXP3 fluorescence intensity [25], elevated effector memory Treg cells expressing HLA-DR [34], enhanced CD4 + CD25+CD127- Treg cells [36], and decreased dysfunctional CRTH2 + Treg cells [33]. These immunological outcomes were secondary endpoints in all studies and were assessed using different methods and at different time points. None of the studies formally examined whether the observed immunological changes contributed to the clinical outcomes. Therefore, these findings are best interpreted as supporting a possible immunomodulatory effect of vitamin D, rather than as evidence that this effect was responsible for the reported clinical differences.

### Endogenous vitamin D level role in AIT efficacy

#### Study characteristics

The 5 studies reviewed evaluated the relationship between endogenous vitamin D levels and AIT clinical efficacy in patients with allergic rhinitis and/or asthma. Of the 5 studies, 2 were post-hoc analyses [40, 44], 2 were observational cohort studies [42, 43], and 1 was a cross-sectional study [41]. Four of the studies included children [40, 42–44], and only 1 included adult patients [41]. All patients were diagnosed with allergic rhinitis and/or asthma, sensitized to HDM in 4 of the studies [40, 42–44], and to various pollens, including weed pollen, grass pollen, and tree pollen in the remaining study [41]. The sensitizations were assessed using skin prick tests and/or sIgE measurement. The types of AIT were represented by SCIT in 3 of the studies [40–42] and SLIT in 2 of the studies [43, 44], one of which opted for allergoid AIT [44].

The studies demonstrated variability in how vitamin D levels were defined and categorized, with different cut-off values and group classifications, mostly into vitamin D sufficiency and insufficiency. A vitamin D level > 30 ng/ml was classified as vitamin D sufficiency in 2 of the studies [40, 41], while 1 defined sufficiency as > 20 ng/ml [42]. Other cut-off values used were 22.25 ng/ml [43], respectively 27 ng/ml [44]. Apart from the vitamin D sufficiency and vitamin D insufficiency groups, two of the studies used additional classifications based on vitamin D levels. Respectively, in the study conducted by Jia X et al. [42], the groups were classified as vitamin D deficiency (< 12 ng/ml), insufficiency (12–20 ng/ml) and sufficiency (> 20 ng/ml), and, in the study conducted by Joudi M et al. [41], groups were categorized into vitamin D deficiency (< 10 ng/ml), insufficiency (10–19 ng/ml), suboptimal levels (20–30 ng/ml) and sufficiency (> 30 ng/ml).

Most studies carried out two visits, the first at baseline, before starting treatment, and the second after 6 months [43] or 1 year [40–42, 44]. In the study conducted by Majak P et al. [40], apart from the final 1-year visit, a prior 3-month visit was carried out. Of the 5 studies, 4 reported that higher vitamin D levels are significantly associated with better AIT clinical outcomes [40, 41, 43, 44], using different parameters to measure clinical efficacy, although in one of them the association was limited to some of the outcomes assessed [44]. Improvements in respiratory symptoms were observed, shown by reductions in asthma symptom scores and ICS doses [40], improvements in quality-of-life, symptom burden scores such as the sino-nasal outcome test-22 (SNOT-22), mini rhinoconjunctivitis quality of life questionnaire (MiniRQLQ) [41] and CSMS [43], as well as reductions in medications such as oral antihistamines accompanied by decreased VAS scores [44]. In the remaining study, all vitamin D groups improved significantly, with no differences between them in symptom or medication scores [42].

## Discussion

The role of vitamin D in AIT outcomes for allergic rhinitis and asthma has garnered increasing attention in recent years. AIT is the only disease-modifying treatment and has long-term benefits. Despite its established efficacy, the clinical response to AIT remains heterogeneous, highlighting the need for strategies to optimize treatment outcomes. This variability has prompted increasing interest in adjunctive approaches aimed at enhancing AIT efficacy and durability, among which vitamin D has emerged as a potential immunomodulatory candidate.

This review analyses the role of vitamin D on the clinical outcomes of AIT for allergic rhinitis and asthma, highlighting the potential for enhanced efficacy of both exogenous supplementation and adequate endogenous levels. We identified 13 studies that assess vitamin D-dependent differences in AIT outcomes in patients who received vitamin D during AIT versus patients undergoing AIT alone or AIT with placebo and 5 studies that assessed the correlation of endogenous levels of vitamin D with AIT outcomes.

Overall, the studies were quite heterogeneous, involving both children and adults, different vitamin D supplementation schemes and different monitoring schedules.

Most studies reviewed reported a clinical benefit of vitamin D supplementation during AIT [27–29, 31, 32, 34, 37], with two further studies reporting benefit limited to a subgroup or a single time point [25, 33]. Improvements were observed across various clinical parameters, including symptom and medication scores, as evidenced by decreases in nasal symptoms, nasal or inhaled corticosteroid use, and an increase in asthma control. Several clinical studies have reported that sufficient serum 25-hydroxyvitamin D levels are associated with improved AIT outcomes. Patients with adequate vitamin D have shown greater reductions in symptom and medication scores, as well as enhanced quality-of-life improvements, compared with vitamin D-deficient individuals [40, 41, 43, 44]. In SCIT, vitamin D supplementation has been linked to accelerated clinical improvement. Similar trends have been observed in SLIT, where baseline vitamin D sufficiency predicted more favorable responses.

Only three of the reviewed studies concluded no benefit in AIT clinical outcomes by supplementing with vitamin D. In the study conducted by Van Hemelen D [26], an explanation could be the fact that a lack of significant clinical improvement was also observed in the AIT + placebo group, therefore making it difficult to draw a definite conclusion on the significance of supplementing with vitamin D. This study also used calcitriol, as did the study by Sudiro M et al. [36], and both reported no added benefit, whereas the studies using vitamin D3 or D2 showed mixed results; the formulation could therefore have contributed to these results, although a clear statement cannot be made. Although the study conducted by Heine G et al. [30] did not show differences in clinical parameters between groups, immunological improvements favoring the AIT + vitamin D group were noted.

Despite reporting clinical outcomes, only a small subset of the reviewed studies also quantified immunological changes during AIT in the context of vitamin D supplementation. Some of the findings included reductions in specific IgE levels, increases in IL-10, and modulation of Treg cells, including enhanced cell numbers and function. However, inconsistencies in IgG4-related findings and limited exploration of the same immunological markers within the studies make it difficult to form a clear conclusion and warrant further investigation. None of the studies examined whether these immunological changes contributed to the observed clinical differences. Therefore, they cannot be considered evidence of a synergistic effect between vitamin D and AIT.

Endogenous vitamin D levels also seem to play a role in the efficacy of AIT for allergic rhinitis and/or asthma, as most of the reviewed studies concluded that patients with sufficient vitamin D levels reported superior clinical outcomes of AIT compared to those with insufficient or deficient levels. Nevertheless, the considerable variability in defining vitamin D sufficiency, insufficiency and deficiency, given the different cut-off values across studies, represents a limitation of this study, as does the small number of studies, which complicates direct comparisons of findings regarding vitamin D’s role in enhancing AIT efficacy.

Most clinical studies to date have primarily evaluated outcomes over short- to intermediate-term follow-up periods, often limited to the active phase of allergen immunotherapy. Consequently, it remains unclear whether vitamin D supplementation can influence the long-term persistence of immune tolerance following the discontinuation of allergen immunotherapy.

Two distinct clinical questions should be considered. First, correction of established vitamin D deficiency is appropriate on general medical grounds, regardless of whether a patient is undergoing AIT. Second, a different question is whether vitamin D supplementation should be used specifically as an adjunct to improve the efficacy of AIT, including in patients with sufficient vitamin D levels. The available evidence does not adequately adress the latter question. Most studies included participants who were vitamin D deficient or insufficient at baseline; therefore, the observed clinical benefits may, at least in part, reflect correction of an underlying deficiency rather than a specific synergistic effect of vitamin D on the immunological mechanisms or clinical efficacy of AIT. Consequently, whether vitamin D supplementation provides an additional benefit beyond correction of deficiency, particularly in vitamin D-sufficient patients, remains uncertain.

One of the main concerns of our review is that many of the trials involved small sample sizes, which reduced statistical power and increased the possibility of biased outcomes. Due to the significant variability across studies, it is challenging to generalize the findings. The limitations of our analysis revolve around differences in the clinical scores and laboratory parameters used in each study, doses, formulations, and administration durations of vitamin D in each study, the different AIT types (SCIT and SLIT), and the different age groups.

As this was a structured narrative review, we did not perform a formal study-level risk-of-bias or certainty-of-evidence assessment. The findings should therefore be viewed as an exploratory synthesis of the available literature rather than as a definitive estimate of treatment effect. Given the limited number of available studies, three conference abstracts and one doctoral thesis chapter were also included as supplementary sources, although their findings were interpreted with greater caution than those from full-length peer-reviewed clinical studies. In addition, restricting the search to English-language publications may have introduced some degree of language bias.

In order to be able to draw clear conclusions about the role of vitamin D on AIT efficacy, more studies are necessary. Standardization of vitamin D regimens, including doses, formulations, administration routes, and treatment duration, as well as studies on the same age groups, supplementing with the same AIT type for the same allergens, evaluating at standardized end-points with consistent clinical parameters, is necessary to enable direct comparison of clinical outcomes within studies. Also, further research is needed to better define vitamin D cut-off values and to define vitamin D sufficiency, insufficiency and deficiency.

Future trials should aim to clarify the optimal serum vitamin D level for AIT responsiveness, the timing of supplementation (before vs. during treatment), and the most effective dosing regimens. Stratified analyses based on genetic polymorphisms in the VDR or enzymes regulating vitamin D metabolism may also identify subgroups more likely to benefit. Longitudinal studies are warranted to evaluate whether vitamin D supplementation not only improves short-term symptom control but also enhances the long-term disease-modifying potential of AIT, including prevention of new sensitizations or asthma progression.

## Conclusions

Current evidence suggests that adequate vitamin D status, and in some studies vitamin D supplementation, may be associated with better clinical or immunological responses to AIT in patients with allergic rhinitis and/or asthma. However, the available evidence remains difficult to interpret because of substantial heterogeneity in study design, sample size, vitamin D formulations and dosing regimens, definitions of vitamin D status, and clinical endpoints. The absence of standardized outcome measures and, in our review, a formal risk-of-bias assessment further limits the strength of the conclusions that can be drawn.

The studies evaluating endogenous vitamin D status were either observational or based on post-hoc analyses, limiting their interpretation to associations rather than causal relationships. Similar caution applies to the reported immunological findings. Although changes in IL-10, allergen-specific immunoglobulins, and regulatory T-cell phenotypes were reported, none of the studies examined whether these changes were responsible for the clinical outcomes observed. They should therefore be regarded as accompanying immunological findings, rather than as evidence that vitamin D and AIT act synergistically in a clinically meaningful way.

Correcting an established vitamin D deficiency remains appropriate for general medical reasons, including in patients undergoing AIT. However, the available evidence is still insufficient to support vitamin D supplementation specifically as a strategy to enhance AIT efficacy or to recommend routine vitamin D screening for this indication. Similarly, there is currently insufficient evidence to recommend routine vitamin D screening solely for the purpose of optimizing AIT outcomes. Further well-designed, placebo-controlled studies using standardized protocols and predefined clinical outcomes, with patients stratified according to baseline vitamin D status, are needed to determine whether vitamin D has a relevant adjunctive role in AIT and to identify the patients most likely to benefit.

## Supplementary Information

Below is the link to the electronic supplementary material.

Supplementary Material 1

## Data Availability

No datasets were generated or analysed during the current study.
