# Supplementary material for: Vitamin D status and supplementation as potential modifiers of allergen immunotherapy outcomes in respiratory allergy: A structured narrative review
Source: Immunol Res. 2026 Oct 2;74(1):119. doi: 10.1007/s12026-026-09854-3 (PMC13633359; doi:10.1007/s12026-026-09854-3)
Supplement: Supplementary file 1 [file 12026_2026_9854_MOESM1_ESM.docx]

Supplementary Table S1 – Complete Search Strategy

| Database | Search Field | Complete Search Strings | Date of Last Search | Records Retrieved |
| --- | --- | --- | --- | --- |
| PubMed | MeSH + Title/Abstract | ( "Vitamin D"[Mesh] OR "vitamin D"[Title/Abstract] OR "vitamin D2"[Title/Abstract] OR "vitamin D3"[Title/Abstract] OR cholecalciferol[Title/Abstract] OR ergocalciferol[Title/Abstract] OR calcifediol[Title/Abstract] OR calcidiol[Title/Abstract] OR calcitriol[Title/Abstract] OR "25-hydroxyvitamin D"[Title/Abstract] OR "25(OH)D"[Title/Abstract] ) AND ( "Desensitization, Immunologic"[Mesh] OR "Sublingual Immunotherapy"[Mesh] OR "allergen immunotherapy"[Title/Abstract] OR "allergen-specific immunotherapy"[Title/Abstract] OR "allergy immunotherapy"[Title/Abstract] OR "immunotherapy, allergen"[Title/Abstract] OR "specific allergen immunotherapy"[Title/Abstract] OR "specific immunotherapy"[Title/Abstract] OR "subcutaneous immunotherapy"[Title/Abstract] OR "sublingual immunotherapy"[Title/Abstract] OR SCIT[Title/Abstract] OR SLIT[Title/Abstract] OR desensitization[Title/Abstract] OR (allerg*[Title/Abstract] AND immunotherap*[Title/Abstract]) ) AND ("2005/01/01"[Date - Publication] : "2026/08/10"[Date - Publication]) AND English[Language] | 10^th^ of August | 126 |
| Scopus | TITLE-ABS-KEY | TITLE-ABS-KEY ( ( "vitamin D" OR "vitamin D2" OR "vitamin D3" OR cholecalciferol OR ergocalciferol OR calcifediol OR calcidiol OR calcitriol OR "25-hydroxyvitamin D" OR "25(OH)D" ) AND ( "allergen immunotherapy" OR "allergen-specific immunotherapy" OR "allergy immunotherapy" OR "immunotherapy, allergen" OR "specific allergen immunotherapy" OR "specific immunotherapy" OR "subcutaneous immunotherapy" OR "sublingual immunotherapy" OR SCIT OR SLIT OR desensitization OR (allerg* W/3 immunotherap* ) ) ) AND PUBYEAR > 2004 AND PUBYEAR < 2027 AND PUBYEAR > 2004 AND ( LIMIT-TO ( LANGUAGE,"English" ) ) | 10^th^ of August | 318 |
| Web of Science Core Collection | Topic | ("vitamin D" OR "vitamin D2" OR "vitamin D3" OR cholecalciferol OR ergocalciferol OR calcifediol OR calcidiol OR calcitriol OR "25-hydroxyvitamin D" OR 25OHD) AND ("allergen immunotherapy" OR "allergen-specific immunotherapy" OR "allergy immunotherapy" OR "allergen specific immunotherapy" OR "specific immunotherapy" OR "subcutaneous immunotherapy" OR "sublingual immunotherapy" OR SCIT OR SLIT OR desensitization | 10^th^ of August | 148 |
| ScienceDirect – Search 1 | Title, abstract, keywords | ("vitamin D" OR cholecalciferol OR calcifediol OR calcitriol) AND ("allergen immunotherapy" OR "allergen-specific immunotherapy" OR "subcutaneous immunotherapy" OR "sublingual immunotherapy") | 10^th^ of August | 6 |
| ScienceDirect – Search 2 | Title, abstract, keywords | ("vitamin D" OR cholecalciferol OR calcifediol OR calcitriol) AND ("allergy immunotherapy" OR "specific immunotherapy" OR SCIT OR SLIT) | 10^th^ of August | 8 |
| ScienceDirect – Search 3 | Title, abstract, keywords | ("vitamin D2" OR "vitamin D3" OR "25-hydroxyvitamin D" OR "25(OH)D") AND ("allergen immunotherapy" OR "allergen-specific immunotherapy" OR "subcutaneous immunotherapy" OR "sublingual immunotherapy") | 10^th^ of August | 1 |
| ScienceDirect – Search 4 | Title, abstract, keywords | ("vitamin D2" OR "vitamin D3" OR "25-hydroxyvitamin D" OR "25(OH)D") AND ("allergy immunotherapy" OR "specific immunotherapy" OR SCIT OR SLIT) | 10^th^ of August | 9 |
| Cochrane Library | MeSH + title, abstract, keywords | #1 MeSH descriptor: [Vitamin D] explode all trees  #2 MeSH descriptor: [Cholecalciferol] explode all trees  #3 MeSH descriptor: [Ergocalciferols] explode all trees  #4 ("vitamin D" OR "vitamin D2" OR "vitamin D3" OR cholecalciferol OR ergocalciferol OR calcifediol OR calcidiol OR calcitriol OR "25-hydroxyvitamin D" OR "25 hydroxyvitamin D" OR 25OHD OR "25(OH)D"):ti,ab,kw  #5 ("allergen immunotherapy" OR "allergen-specific immunotherapy" OR "allergen specific immunotherapy" OR "allergy immunotherapy" OR "specific immunotherapy" OR "subcutaneous immunotherapy" OR "sublingual immunotherapy" OR SCIT OR SLIT OR desensitization OR "desensitization therapy":ti,ab,kw  #6 MeSH descriptor: [Calcifediol] explode all trees  #7 MeSH descriptor: [Calcitriol] explode all trees  #8 MeSH descriptor: [Desensitization, Immunologic] explode all trees  #9 #1 OR #2 OR #3 OR #4 OR #6 OR #7  #10 #5 OR #8  #11 #9 AND #10 | 12^th^ of August | 37 |
